# Ultrasound Detected Myokymia Is Associated with Increased Duration of Hospitalization and Healthcare Cost After Rattlesnake Bite in Arizona

**DOI:** 10.3390/toxins18090391

**Published:** 2026-09-10

**Authors:** Farshad Mazda Shirazi, Srikar R. Adhikari, Vance G. Nielsen, Geoffrey Thomas Smelski

**Affiliations:** 1 Department of Medical Pharmacology, University of Arizona, Tucson, AZ 85721, USA; mshirazi@aemrc.arizona.edu; 2 Department of Emergency Medicine, University of Arizona, Tucson, AZ 85721, USA; sadhikari@aemrc.arizona.edu; 3 Department of Anesthesiology, University of Arizona, Tucson, AZ 85721, USA; 4 College of Pharmacy, University of Arizona, Tucson, AZ 85721, USA; gsmelski@pharmacy.arizona.edu

**Keywords:** snake bite, venom, rattlesnake, neurotoxicity, fasciculations, myokymia, creatine kinase, length of stay, healthcare cost

## Abstract

Neurotoxic envenomation by rattlesnakes has been of great concern throughout the Americas and in Arizona. In addition to clinical weakness, patients sometimes display myokymia, a disordered fasciculation of skeletal muscle, which is mediated by Mojave toxin. As neurotoxic venoms have been associated with increased release of creatine kinase (CK) activity and concordant increases in length of stay (LOS) in the hospital, it was retrospectively determined if point-of-care ultrasound detected myokymia at the bite site was associated with increased CK activity, LOS, and healthcare cost compared to rattlesnake envenomations not associated with myokymia. The Arizona Poison and Drug Information Center patient database was queried for patients with point-of-care ultrasound exam results at or near the bite site from 2017 to 2021. Using point-of-care ultrasound to detect subclinical myokymia, thirty-seven patients that met inclusion criteria were determined to have myokymia (n = 16) and/or not display it (n = 21). Patients with myokymia had twice the CK activities, double the LOS, and two-fold the healthcare costs of patients without myokymia detected. These findings serve as a rationale for future investigations to link ultrasound detected myokymia with patient outcomes.

## 1. Introduction

Neurotoxicity has been a medically important concern following venomous snake bite worldwide, with manifestations ranging from regional to generalized muscular weakness, disordered muscular fasciculations or myokymia, and potentially apneic death [1,2,3,4,5,6,7,8,9,10,11,12,13,14]. The components of neurotoxic venom may include postsynaptic membrane neuromuscular junction binding proteins such as three-finger toxins [5,6] or phospholipase A_2_ (PLA_2_) that may affect both presynaptic and postsynaptic neuromuscular junctions [6]. In the case of rattlesnake envenomation in the Americas, species with venom most likely containing neurotoxic PLA_2_ have been documented to cause both weakness and/or myokymia that has not always been attenuated by antivenom administration [7,8,9,10,11,12,13]. Specifically, envenomation by California rattlesnakes clearly identified as or thought likely to be Mojave rattlesnakes (*Crotalus scutulatus scutulatus*) in the 1990s resulted in neurological symptoms that would initially abate with antivenom administration but then recur, return and again be attenuated with antivenom, or not be abrogated at all after antivenom in case reports [7,8,9,10]. A statewide poison control system retrospective study yielded similar results [11]. However, it is of interest that a review of 3440 cases of rattlesnake bite in Arizona from 1999 to 2020 demonstrated that not one person required mechanical ventilation, and it was estimated that several hundred to over a thousand of these bites involved Mojave rattlesnakes [14]. It has been noted to be difficult to discern neurotoxic from hemotoxic Mojave rattlesnakes based on physical appearance [14,15,16,17]. The location and elevation in southern Arizona are found to be the best associated determinants for predicting where the neurotoxic (type A) or hemotoxic (type B) types of venoms are found, as well as the zones of where snakes phenotypically displaying blended type A/type B venom are encountered [15,17,18]. The β-neurotoxin in Mojave rattlesnake venom, known as Mojave toxin (MoTX), is a heterodimer with an acidic and a basic PLA_2_ subunit that in early studies demonstrated irreversible diaphragm paralysis in a murine model [19,20]. Subsequent investigation in rodent brain demonstrated that this was due to noncompetitive binding of the calcium channel dihydropyridine receptor binding [21]. Recent in vitro, cell-based investigation has demonstrated that MoTX interacts with several proinflammatory pathways (e.g., activation of mitogen-activated protein kinases, activation of nuclear factor kappa-light-chain-enhancer of activated B cells, upregulation of unfolded protein response) to cause apoptotic and ferroptotic cell injury and death [22]. Moving from the molecular to geographic consideration, several species of rattlesnake in the United States that produce neurotoxic venom containing MoTX or MoTX-like toxins include the following: tiger rattlesnake (*Crotalus tigris*) [23], Grand Canyon rattlesnake (*Crotalus oreganus abyssus*) [24], Southern Pacific rattlesnake (*Crotalus helleri*) [25], timber rattlesnake (*Crotalus horridus horridus*) [26], and the canebrake rattlesnake (*Crotalus horridus atricaudatus*) [27]. In summary, envenomation by the aforementioned rattlesnake species producing neurotoxic venom is of significant medical importance and involve MoTX-associated symptoms.

Of interest, rattlesnake venoms containing MoTX or MoTX-like toxins are also associated with myotoxicity and concordant increases in circulating blood creatine kinase [CK] activity, with peak values varying from thousands to millions of U/L after rattlesnake envenomation [7,8,12,13,18,24,27]. These reports describe patients with very large CK activity release that died [13,27]. Also, such patients had prolonged hospitalizations between 3 and 28 days with or without time in the intensive care unit [7,8,12,13,18,24,27]. Surprisingly, there is an absence of reports for rattlesnake bites from commonly encountered species without MoTX, such as *Crotalus atrox*, detailing significant increases in circulating CK activity. While there is no disputing that *C. atrox* venom and similar rattlesnake venoms cause local tissue damage, including muscle injury, it is possible that the CK activity observed in venoms with MoTX represents systemic myotoxicity beyond the bite site. In regions distant from Arizona, such as Turkey and Africa, increases in circulating CK activity as a measure of injury has been associated with an increase in hospitalization time (length of stay, LOS) and a predictor of severity of symptoms after viper envenomation by species such as *Macrovipera lebetina* and *Vipera barani* [28,29]. In the southeastern coast of China (Fujian), rhabdomyolysis caused by snake bite resulted in circulating CK activity >3000 U/L that was associated with a two-fold increase in the length of hospital stay with the highest incidence from envenomation by *Protobothrops mangshanensis* [30], with similar increases in hospital stay noted in another southern region of China (Nanning) where peak CK >500 U/L was associated with a 2-week to 2-month hospitalization most notably from *Naja* species [31]. Taken as a whole, these studies demonstrate that local and systemic myotoxicity, as determined by circulating peak CK activity, are associated with complicated medical courses not just after rattlesnake envenomation but also by other species of venomous snakes worldwide.

For the researchers that study and practitioners that treat venomous snake bites, the preceding bodies of information serve as a foundation to formulate prognostic paradigms. Perhaps myokymia and circulating CK activity are linked in cases of rattlesnake envenomation in Arizona. The presence of myokymia at the bite site or in muscle groups distant from the bite site after envenomation by rattlesnakes with venom containing MoTX is not always visible [8,9,10,11,12,18,24,25,26]. However, point-of-care (POC) ultrasonic examination of muscle groups at or near the bite site could potentially be performed to detect “subclinical” myokymia as this method has been utilized in this setting [32,33,34]. Another association of importance to evaluate is whether the presence of myokymia after snake bite is associated with prolonged hospitalization and consequent increased healthcare cost. Linking myokymia with a worse outcome, especially myokymia that is not externally visible without ultrasound, would be of great interest.

The goal of the present investigation was to retrospectively query the Arizona Poison and Drug Information Center snake bite database to analyze patient data that included several parameters that included POC ultrasonic examinations at or near the bite site. The working hypothesis was that the presence of myokymia would be associated with increased circulating CK activity during hospitalization, and that these two events would be linked to either greater or lesser LOS and healthcare costs.

## 2. Results

### 2.1. Patient Population That Was Envenomed by Rattlesnakes and Had a POC Ultrasonic Exam upon Presentation

Identification of patients with POC ultrasonic data from the snake bite database maintained by the Arizona Poison and Drug Information Center was performed as detailed in the Materials & Methods section subsequently. After applying exclusion criteria, twenty-one patients without myokymia and sixteen patients with myokymia detected with POC ultrasound were identified for analysis. The demographic and envenomation characteristics of the patients studied are contained in Table 1.

There were no statistically significant differences in age or gender between the two groups. Nearly all bites occurred distal to the elbow or knee. The majority of patients presented to the emergency department and received antivenom in under four hours. The two antivenoms administered for rattlesnake bite included either crotalidae polyvalent immune fab-ovine (Fab) (CroFab^®^, BTG International Inc., Conshohocken, PA, USA) or crotalidae immune F(ab′)_2_ (Anavip^®^, Rare Disease Therapeutics Inc., Franklin, TN, USA). Most patients were administered Anavip^®^, a few others were treated with CroFab^®^, and five patients were administered both. These are the two antivenoms approved by the United States Food and Drug Administration to treat rattlesnake envenomated patients. In summary, the two groups were similar in terms of demographics, envenomation injury, and therapy.

### 2.2. Outcome Data After Envenomation

The outcome data of these two groups of rattlesnake envenomed patients are presented in Table 2. There were large and statistically significant differences between the two groups. Peak circulating CK activity was more than two-fold greater in patients determined to have myokymia by POC ultrasound upon presentation than in those without it. Similarly, patients found to have myokymia were noted to have more than twice the hours of hospitalization than patients without myokymia. Lastly, the cost of hospitalization was nearly double the value in patients with myokymia compared to patients lacking this ultrasonic finding. In summary, patients found with myokymia upon presentation to our emergency department had approximately twice the maximum observed circulating CK activity, hospitalization time, and cost of hospitalization of patients without this POC ultrasonically detected sign.

## 3. Discussion

The present investigation completed its stated goal. An association between POC detected myokymia, increased circulating CK activity, hospitalization time, and healthcare cost was identified. While these findings are of interest, other factors need to be evaluated while future studies explore the mechanism(s) linking myokymia with hospitalization time and medical cost. These data are hypothesis-generating and the following paradigms are proposed as possible explanations for the findings of the present investigation.

The first paradigm posits that the increase in circulating CK activity associated with myokymia may indicate that myotoxicity caused by MoTX results in a larger degree of impairment, pain, or other medical complications compared to envenomed patients without myokymia. While this reasoning seems logical, it needs to be noted that muscular fasciculations from a variety of causes results in increased circulating CK activity that would not be associated with greater time of hospitalization or healthcare cost. For example, the administration of succinylcholine, a medication that paralyzes patients for endotracheal intubation, causes clinically noticeable, systemic muscular fasciculations that can increase circulating CK activity 2–4 times baseline values [35,36,37]. Administration of this medication occurs daily in the conduct of anesthetic practice without remarkable increases in hospitalization time or increased healthcare costs. It is important to note that the fasciculations only persist for less than a minute but involve all the skeletal muscle of the patient until the medication is catalyzed by plasma cholinesterase activity [35,36,37]. In contrast, myokymia may or may not be visually observed after rattlesnake envenomation by neurotoxic rattlesnakes, and antivenom administration may or may not terminate clinically apparent myokymia [7,8,9,10,11,12,13,24,25,26,27]. While myotoxicity mediated by MoTX may be responsible for the increase in circulating CK activity observed in Table 2, there may be a significant contribution to this value by persistent fasciculations (subclinical POC ultrasonic or clinically observable) near or distant from the bite site that persist mildly for hours prior to antivenom administration. Of interest, envenomation by a black mamba (*Dendroaspis polylepis*) resulted in several hours of fasciculations likely secondary to fasciculins (three finger toxin, an α-toxin) [38]. The patient was mechanically ventilated, and circulating CK activity was noted to increase to 649–1248 U/L [38]. Both antivenom and atracurium (a nondepolarizing neuromuscular agent) were required to attenuate fasciculations and allow a normal recovery days later [38]. Of interest, in this case it was the fasciculations that were attributed to the rise of circulating CK activity, and the recovery of neurological function was the primary determinant of time of hospitalization and healthcare cost and not myotoxicity per se [38]. In contrast, fasciculation mediated CK activity release is not a confounding factor in the cases of venom inflicting rhabdomyolysis observed elsewhere [28,29,30,31]. Thus, while promising as a potential marker of important systemic myotoxicity, increased circulating CK activity following rattlesnake bite may have a component dependent on fasciculations and not just muscle cellular lysis, making a strong link to increased time of hospitalization and healthcare cost secondary to systemic injury less certain.

Another paradigm that could be suggested is that the myokymia associated with envenomation may more efficiently move venom from the bite site into the regional tissues and systemic circulation. Lymphatic transport of the contents of the interstitial space is a primary mechanism by which venom travels proximally from the bite site through the limb of an envenomed patient [39,40,41]. Critically, skeletal muscle contracture accelerates lymphatic flow [42]. Thus, myokymia detected with POC ultrasound could have been moving venom from the bite site for perhaps hours, involving tissues near the bite site and critically delivering the venom into the systemic circulation more efficiently and with greater quantity compared to envenomation not associated with myokymia. It is also possible that, consistent with this paradigm, greater amounts of venom are delivered in bites from snakes with MoTX or MoTX-like activity present in their venom that is then subsequently systemically absorbed via myokymia. In summary, myokymia may have served as a mechanism for more efficient systemic envenoming, resulting in greater hospitalization time and healthcare cost.

**Figure 1:**
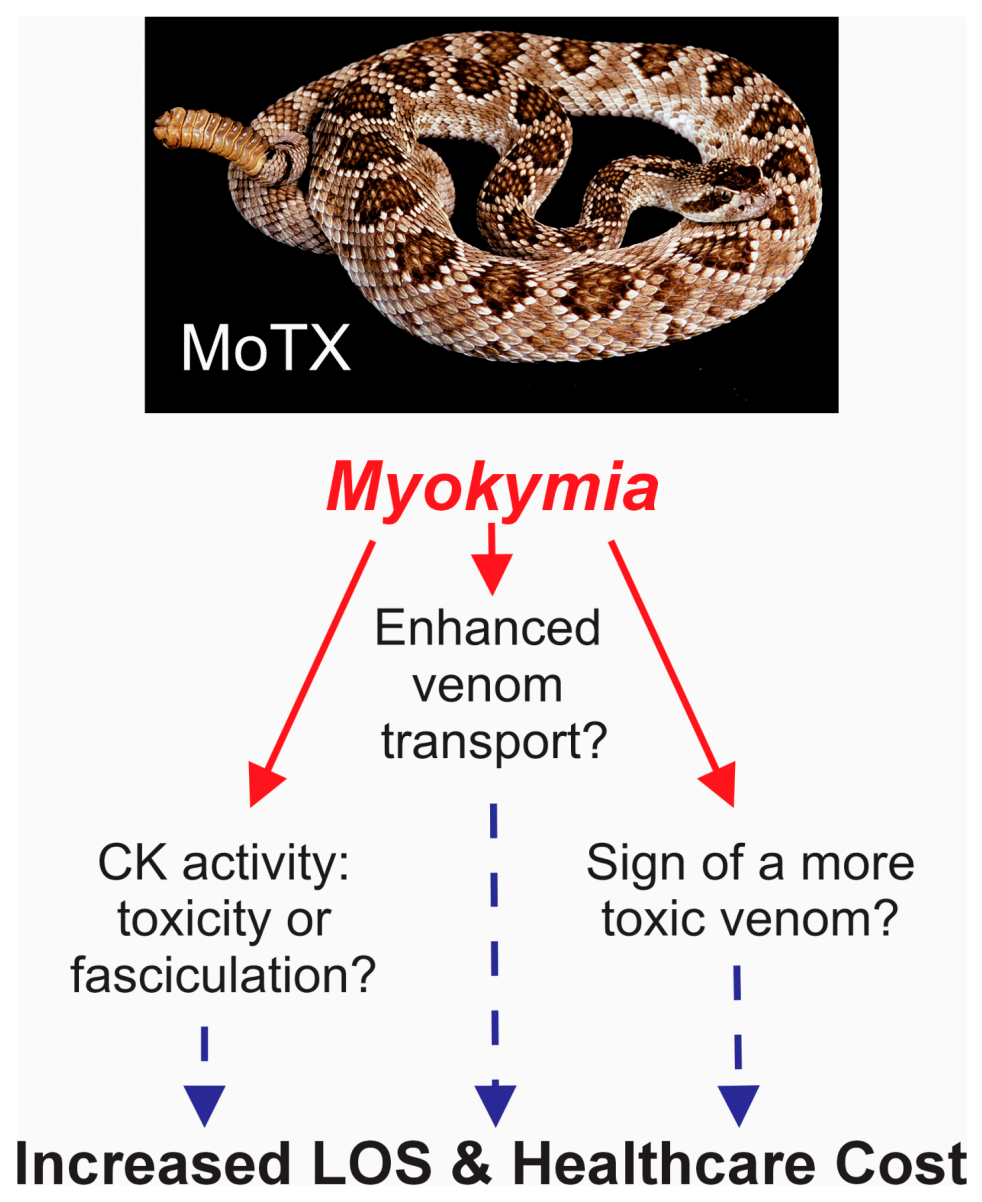
Potential paradigms concerning the association of myokymia following envenomation with a venom containing MoTX or MoTX-like activity and increased length of stay (LOS) and healthcare cost (Mojave rattlesnake depicted).

A third paradigm may be that myokymia serves as a physical sign phenotypically identifying a venom that is more systemically damaging than a venom not associated with it; and, that myokymia per se has little to directly do with increased time of hospitalization and healthcare cost. The MoTX, in conjunction with not just other mostly PLA_2_ in the case of Mojave rattlesnake venom [17] but also with metalloproteinases and serine proteases in the case of other rattlesnakes [23], may result in greater systemic injury than with venom resplendent in metalloproteinases, serine proteases, and PLA_2_ without MoTX as in the case of Western diamondback rattlesnake venom [43]. In conclusion, a summary of these three potential paradigms is depicted in Figure 1.

While the results of the present retrospective investigation are potentially of great interest, the study has limitations observed with all such endeavors. Associations, not relationships, can be determined in order to generate forward thinking hypotheses. In addition to conducting much larger, prospective investigations with POC ultrasonic examination of acutely rattlesnake envenomed patients, greater granularity in concurrent clinical data must be obtained. The precise reasons for patients’ duration of hospitalization (e.g., severe pain, acute kidney injury) and healthcare costs (e.g., prolonged intensive care unit occupancy, dialysis) need to be discerned and associated with incidence and duration of myokymia. Additional investigation could also include studies to determine if antivenom administration should be guided by not just stopping the advance of venom in the affected bitten limb but also by the attenuation or elimination of myokymia regionally and systemically. Multiple outcomes in such investigations could be considered to modify present day therapy to improve patient care. However, at a minimum, the presence of myokymia upon presentation could potentially provide a future prognosis of more prolonged and expensive hospital courses if no therapeutic intervention is found to be effective. Phrased another way, perhaps by the time myokymia can be detected with ultrasound it is too late—enough venom has been released systemically to wreak havoc and result in a worse outcome than observed with venoms without MoTX. In summary, the data of the present investigation serves as the rationale for future investigations to determine the utility of POC ultrasound detected myokymia as a prognostic clinical sign and potential marker of the success of therapeutic interventions.

## 4. Materials and Methods

### 4.1. Rattlesnake Bite Database

The present retrospective study was conducted in accordance with the Declaration of Helsinki, and the protocol was approved by the University of Arizona Institutional Review Board (2108137685) on 20 August 2021. The Arizona Poison and Drug Information Center serves the state of Arizona, excluding Maricopa County. The Center’s standard practice for managing rattlesnake bites includes recommending antivenom for all patients with signs of envenomation. The Center’s records for all cases coded as a rattlesnake bite receiving antivenom were searched between 2017 and 2021 for inclusion of POC ultrasonic exam upon arrival to the Banner University Medical Center emergency department and hospital. The area examined by ultrasound was either at the bite site if there was musculature present (e.g., hand, calf) or at the most proximate area wherein musculature was associated with it (e.g., if a finger or toe, then the hand or foot, respectively). Explicitly, in the case of fingers, there is no muscle present and the hand would be the first place to expect myokymia to occur after envenomation. The exclusion criteria are subsequently presented. A total of fifty patients were identified. Two were excluded as the results of the ultrasonic examination were not documented in the Center’s records. Of those remaining, six were excluded as they were under the age of 18 years; three were excluded as they had been bitten multiple times, bitten on the head, or been bitten on the torso; and, lastly, two were excluded as their records were missing all three of the major outcomes (e.g., circulating CK activity, hours of hospitalization, and cost of healthcare [United States Dollars, $USD]). After applying these exclusion criteria, there were twenty-one patients without myokymia present confirmed by ultrasound, and sixteen patients with myokymia detected by ultrasound remaining for analysis. The data presented with the POC determination of myokymia are a subset of data collected for the Arizona Poison and Drug Information Center as previously published [44]. An abstract presented at a meeting in 2020 [32] may have also included ultrasonic results of some of the patients in the present study and in the aforementioned citation [44]. However, it is unknown if the patients included in the abstract [32], with only two displaying myokymia, were included or excluded secondary to insufficient granular data on the patient level.

Outcome parameters were defined as subsequently presented. The largest CK activity measured during hospitalization was reported to the Arizona Poison and Drug Information Center. Similarly, the number of days of hospitalization reported to the Arizona Poison and Drug Information Center were recorded. Lastly, healthcare cost was defined as the $USD reported to the Arizona Department of Health Services by each hospital. In turn, these data were placed into the Arizona Poison and Drug Information Center records. They reflect the bill from the hospital that was actually paid by the patient or their health insurance provider. No attempt at adjusting the cost to a common year was performed; instead, the cost observed from year to year was reported without adjustment.

The determination of myokymia was retrospectively reported to the database of the Arizona Poison and Drug Information Center following the conduct of the subsequently prospectively conducted protocol. Patient recruitment, informed written consent, and retrospective review of archived ultrasound images was performed after approval by the University of Arizona Institutional review board (Protocol number 1806663505, approved on 19 July 2018 and is ongoing) and was entitled “Sonographic Findings and Subsequent Management of Snakebites.” The patients included in the present study were examined in the time period of 2018–2021. As this was a proof-of-concept study, only a few potentially envenomed patients eligible for antivenom administration were studied over the indicated period of time. In brief, emergency physicians with varying but adequate levels of ultrasound experience performed bedside soft tissue ultrasound following the initial clinical assessment of an envenomed patient. Bedside soft tissue ultrasound examinations were performed at the anatomical site of the snake bite using FDA-cleared ultrasound systems equipped with a high-frequency broadband linear array transducer. Gray-scale (B-mode) imaging was obtained in both longitudinal and transverse planes. Still images and video clips of the affected soft tissues, including the skin, subcutaneous tissue, fascial planes, and underlying musculature, were acquired and stored in the institutional point-of-care-ultrasound (POCUS) image archive as part of routine clinical documentation. The ultrasound examination extended from the bite site proximally to the most proximal extent of sonographic tissue involvement. The sonographic leading edge of soft tissue abnormalities was compared with the clinically marked leading edge of swelling at the bite site. Archived ultrasound images and video clips were reviewed for predefined sonographic findings, including myokymia, by one of the senior investigators with significant ultrasonic experience. Myokymia was defined by disordered muscular fasciculations as diagnosed by the senior investigator.

Both the contemporaneous bedside ultrasound report and retrospective review of the archived ultrasound clips were used to determine the final classification. The classification was confirmed retrospectively by ultrasound faculty and fellows with 5–15 years of POCUS experience. Outcome data were masked during retrospective image assessment to minimize potential interpretation bias. Uncertain or equivocal cases were resolved through group discussion and consensus among the reviewers.

### 4.2. Statistical Analyses and Graphics

Data are presented as mean ± SD or median and (1st, 3rd) quartiles. A commercially available statistical program was used for dataset analyses with unpaired, two-tailed Student’s *t*-test, chi-square, or Mann–Whitney Rank Sum Test if data were found to fail tests for normality (Shapiro–Wilk) and equal variance (Brown–Forsythe) SigmaStat 3.1; Systat Software, Inc., San Jose, CA, USA). Graphics were generated with commercially available programs; Origin 2026, OriginLab Corporation, Northampton, MA, USA; and CorelDRAW 2024, Alludo, Ottawa, ON, Canada). *p* < 0.05 was considered significant.

## Figures and Tables

**Table 1:** Demographics, bite site, and antivenom administration data.

Parameter	Myokymia	No Myokymia
Patient number	16	21
Age (years)	57.9 ± 18.7	49.9 ± 19.6
Male:Female Ratio	14:2	15:6
Finger/Hand Bite	7	11
Toe/Foot/Ankle Bite	3	7
Calf/Thigh Bite	6	2
Arm Bite	0	1
Antivenom in <4 h	14 of 16	18 of 21
Anavip^®^	11 patients	18 patients
CroFab^®^	1 patient	2 patients
Anavip^®^ + CroFab^®^	4 patients	1 patient

Data are presented as raw data or as mean ± SD.

**Table 2:** Peak CK activity, LOS, and healthcare cost.

Outcomes	Myokymia	No Myokymia
Peak CK Activity (U/L)	276 (165, 1374) [14] *	125 (94, 202) [18]
LOS (days)	2.6 (1.8, 4.7) [16] **	1.2 (0.9, 2.0) [18]
Healthcare Cost ($USD)	$127,464 (81,516, 190,875) [16] ***	$65,932 (48,952, 133,467) [18]

Data are median (1st, 3rd quartiles). [] are number of patient entries for the group available for statistical analysis. Myokymia was determined with POC ultrasound. * *p* = 0.006 vs. No Myokymia; ** *p* = 0.004 vs. No Myokymia; and, *** *p* = 0.032 vs. No Myokymia.

## Data Availability

The original contributions presented in the study are included in the article and further inquiries can be directed to the corresponding author.
